# The Higher Proportion of Men with Psoriasis Treated with Biologics May Be Explained by More Severe Disease in Men

**DOI:** 10.1371/journal.pone.0063619

**Published:** 2013-05-15

**Authors:** David Hägg, Marie Eriksson, Anders Sundström, Marcus Schmitt-Egenolf

**Affiliations:** 1 Dermatology and Venereology, Public Health and Clinical Medicine, Umea University, Umea, Sweden; 2 Umeå School of Business and Economics (USBE), Department of Statistics, Umea University, Umea, Sweden; 3 Centre for Pharmacoepidemiology (CPE), Karolinska Institutet, Karolinska University Hospital, Stockholm, Sweden; INSERM-Université Paris-Sud, France

## Abstract

**Objectives:**

Moderate to severe psoriasis, once regarded as merely a skin disease, is today seen as an inflammatory systemic disease. The sex ratio of the prevalence of psoriasis is balanced. In recent years several reports have documented that men receive more systemic or UV treatment than women, and different hypotheses were made. In PsoReg, the national registry for systemic treatment of psoriasis in Sweden, we have, like other European registries, observed a predominance of men (59%), especially of men treated with biologics (63%). Biologics are a relatively new group of very effective but high-priced drugs. The objective of this study was to analyse if women are discriminated by not having the same access to the high-priced biologics.

**Design:**

Population based cohort study using data from a nationwide quality register of psoriasis patients.

**Population:**

2294 patients with moderate to severe psoriasis receiving systemic treatment from a specialist in dermatology.

**Main Outcome Measures:**

Time to initiation of biologic treatment. A multiple Cox proportional hazard’s regression was performed, with time to initiating a biologic treatment as the outcome in order to assess the independent role of the patient’s sex in initiating such therapy. The psoriasis severity was defined as a time-varying variable.

**Results:**

Men had more severe psoriasis than women according to the Psoriasis Area and Severity Index (PASI), regardless of age at enrolment, and throughout the study period. The analysis in the multiple Cox regression show that age, psoriasis severity and psoriasis arthropathy were relevant factors for initiating biologic therapy, whereas sex is not.

**Conclusions:**

Although as many women as men are believed to suffer from psoriasis, men seem to be more severely affected by psoriasis. The asymmetry in allocation of biologic therapy thereby probably reflects the differing disease activity between the sexes, and is not a discrimination against women per se.

## Introduction

Psoriasis is a chronic inflammatory skin disease with a prevalence of 2–3% in the Swedish population. About 10–30% of the patients who are suffering from psoriasis may also be affected from psoriatic arthritis (PsA). Moderate to severe psoriasis is associated with comorbidities such as depression, metabolic syndrome and cardiovascular diseases [1]–[4]. The prevalence of psoriasis is estimated to be equal among men and women [5], [6].

Mild psoriasis can be treated with topical treatments only, while moderate to severe psoriasis requires phototherapy or systemic treatment. The dominant systemic treatment is methotrexate. However, many patients need a high-priced alternative, *i.e.* biologic treatment. Biologic agents were licensed for treatment of psoriasis in Sweden 2004 and have been shown to be highly effective in treating moderate to severe psoriasis [7], [8].

PsoReg, the Swedish registry for systemic psoriasis treatment was established in 2006 as a longitudinal prospective database to improve psoriasis care and to analyse the safety and effectiveness of systemic psoriasis treatments, especially biologics [9]. PsoReg covers over 65% of the biologic treatments and about 45% of the overall systemic treatment for moderate to severe psoriasis in Sweden.

The majority of patients registered in PsoReg are men (59.2%). This is in line with other European registries in the PsoNet group, showing a notable dominance of men with 68% in the Netherlands, 67% in Italy, 66% in Denmark, 63% in Spain and 60% in Germany [10]. There is an active discussion whether women are discriminated by not receiving the same quantity of high-priced drugs as men in several fields of medicine [11], [12]. Previous studies indicate that men receive systemic or UV treatment for psoriasis in greater extent than women [13], [14]. This study was initiated to analyse whether men more often receive high-priced systemic treatment in form of biologics, also when taking other factors such as disease severity into account.

## Materials and Methods

### Ethics Statement

This research was done in adherence to the Declaration of Helsinki. Patients were recruited after that informed consent was obtained. Both data and consent was gathered electronically, to assure an effective logistic in this nationwide project. The Umeå Ethical Review Board, Sweden approved the project and the procedure for patient consent.

### Study Population

The inclusion criteria for PsoReg are a diagnosis of moderate to severe psoriasis and initiated systemic treatment. Only patients treated by specialists in dermatology are eligible. Patients were registered at local, regional and university hospitals as well as at private practices and treatment centres run by the psoriasis patient organisation (PSO). For each visit, the clinician registers the patient’s weight, treatments, and the psoriasis severity in form of the Psoriasis Area and Severity Index (PASI), which is the most widely used tool to assess the severity of psoriasis [15], [16]. The PASI measures the severity of the skin condition (scaliness, redness and thickness) and the extent of the affected area (head, body, arms and legs). The assessment is then converted into a score that ranges between 0 (no active disease) and 72 (theoretical maximal disease activity) [17].

**Figure 1:**
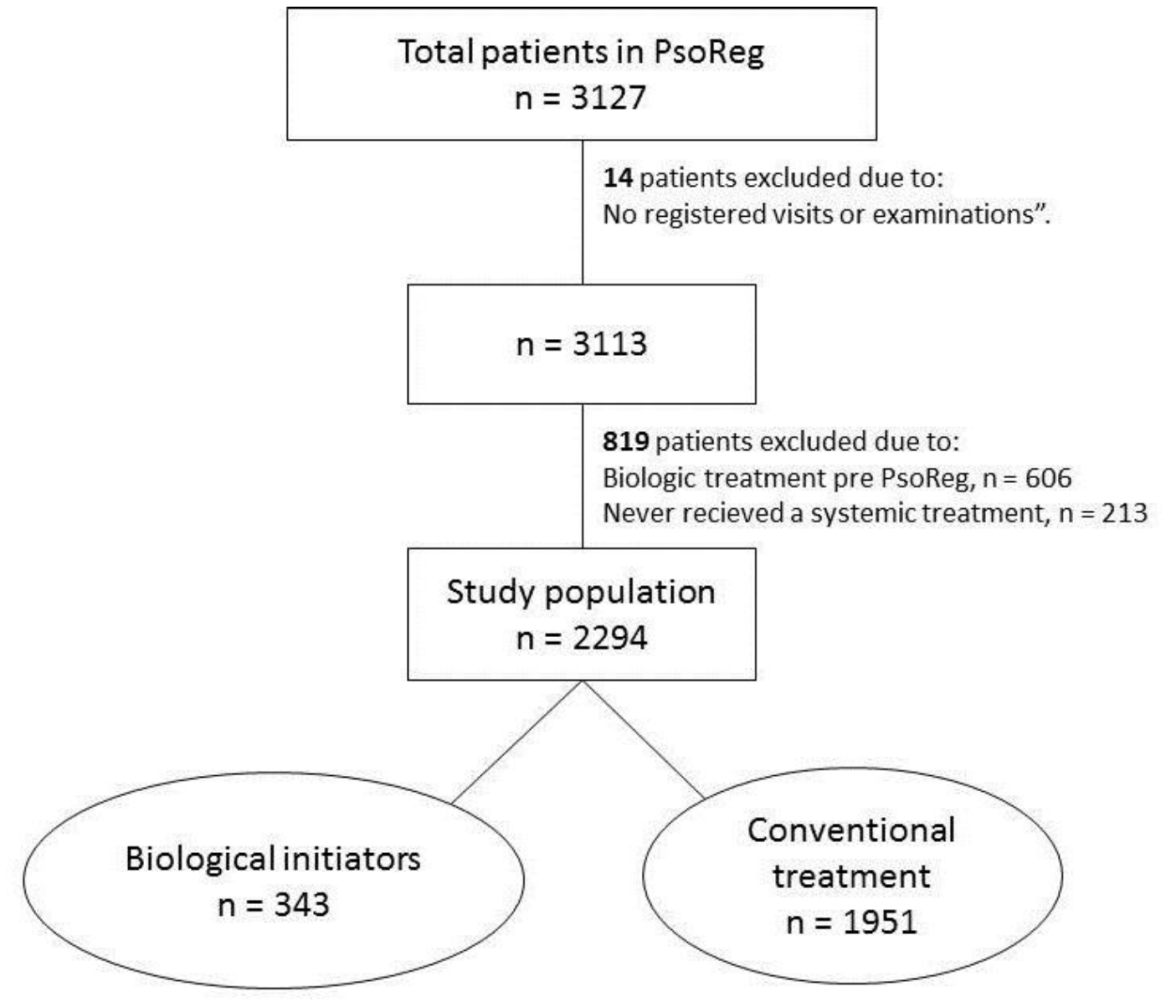
Study population.

At the time of the data extraction in October 2011, there were 3127 patients registered. The study period for this analysis is from February 2006 (the start of the register) through October 2011, and 2294 patients who had not been treated with a biologic agent before enrolment in the register were finally included. In total 343 patients initiated a biologic therapy while 1951 patients remained on conventional treatment during the study period; all study subjects had at least one registered value of PASI during follow-up (figure 1). The 606 patients who were excluded in the analysis because they had received biological treatment before registration in PsoReg were mostly men (65.8%).

### Outcome and Co-variate Definitions

The outcome variable was time to initiation of a biologic agent during follow-up. The number of days to treatment initiation was calculated for each patient starting at enrolment in the register until the initiation of biologic treatment, death, or the end of the study period, whichever occurred first. Patients could enter and finish the study at different points in time and consequently contribute with various numbers of days of follow-up to the analysis.

Two demographical variables were included in the study, sex and age. Age was categorised into ten years’ intervals. Body Mass Index (BMI) was grouped into four different categories using the definition of the World Health Organization (WHO). A BMI below 18.5 was classified as underweight, BMI between 18.5 and 24.9 as normal weight, BMI between 25.0 and 29.9 as overweight and 30 and above as obese. Whether or not the patient is suffering from psoriatic arthritis (PsA) may influence the decision of initiating biologic treatment, reason for which PsA was included in the analysis.

PASI was used to quantify the disease activity. The PASI assessment was categorized into three different groups, by the 33^rd^ and the 66^th^ percentiles of the observed data at start of follow-up, leading to the three categories of low (0.0–4.0), medium (4.1–9.3) and high PASI (9.4+).

### Statistical Methods

Baseline characteristics were assessed for the whole study population. Differences in PASI–scores at baseline between men and women were analysed by a non-parametric Wilcoxon Two-Sample test. Similarly we tested if there were any differences between men and women at time of biological initiation. Differences in proportions or continuous variables were tested by Chi-square tests and Student’s t-test, respectively.

Time to initiation of biologic treatment was analysed by multiple Cox regression stratified by calendar year. Variables included in the model were sex, BMI and PsA, age and the category of PASI, of which the two latter were defined as time varying. Patients were censored at death or emigration. To show how the effects were modified when implementing a multiple model, univariate Cox regressions were performed for each covariate in order to illustrate such differences. Associations between the covariates and outcome are presented by hazard ratios (HR) with 95% confidence intervals (CI). The level of significance was chosen as 0.05. Statistical analyses were performed in SAS version 9.3 for Windows.

## Results

The total study population of 2294 patients consisted of 936 women (40.8%) and 1358 men (59.2%). At inclusion men (mean: 52.2) were younger than women (mean: 56.0), p<0.001. Furthermore, men had on average a higher BMI (27.8) than women (27.1) at inclusion (baseline), p = 0.002. Men had also a higher PASI value (median 7.3) at baseline compared to women (median 5.1); Wilcoxon Two-Sample test: p<0.001.

There were no differences between men and women when we compared percentage of obese patients (p = 0.91) or disease duration (p = 0.23) at baseline (table 1). We also examined whether there was any difference regarding initiation of biologic treatment between women in childbearing age and the corresponding age in men, but no difference was found (data not shown).

**Table 1:** Differences between men and women at baseline: α – t-test, β – Wilcoxon Two-Sample test, γ – Chi-square test.

	Men, n = 1358	Women, n = 936	P-value	Total study population, n = 2294
**Age (Years)**				
Mean (SD)	52.2 (14.8)	56.0 (15.5)	<.001^α^	53.8 (15.2)
**Time of follow-up (months)**				
Mean (SD)	12.3 (12.8)	13.0 (12.9)	0.23^α^	12.6 (12.9)
**Disease duration at start of follow-up (years)**				
Mean (SD)	25.6 (14.7)	27.8 (16.5)	0.23^α^	26.5 (15.5)
**PASI**				
Median (IQR)	7.3 (3.6–12.4)	5.1 (2.7–9.9)	<.001^β^	6.3 (3.1–11.4)
33^rd^ and 66^th^ percentiles	4.7 and 10.4	3.2 and 7.5		4.4 and 9.4
**BMI**				
Mean (SD)	27.8 (4.8)	27.1 (5.8)	0.002^α^	27.5 (5.2)
% obese (BMI ≥30)	27.2	27.5	0.91^γ^	27.3

During the study period 127 women (13.6%) and 216 men (15.9%) initiated a biologic treatment. At the time of the biological initiation men had a higher PASI value (median: 12.3) compared to women (median: 9.8), p = 0.001. No other differences between men and women at time of initiating biological treatment were observed (table 2).

**Table 2:** Differences between men and women at time of biological initiation: α – t-test, β – Wilcoxon Two-Sample test, γ – Chi-square test.

	Men, n = 216	Women, n = 127	P-value	Biological initiatiors, n = 343
**Age (Years)**				
Mean (SD)	47.0 (13.7)	49.4 (15.1)	0.13^α^	47.9 (14.3)
**Time of follow-up (months)**				
Mean (SD)	7.5 (10.6)	8.4 (11.5)	0.48^α^	7.8 (10.9)
**Disease duration at start of follow-up (years)**				
Mean (SD)	24.4 (13.6)	27.0 (15.0)	0.09^α^	25.3 (14.2)
**PASI**				
Median (IQR)	12.3 (7.2–18.4)	9.8 (4.8–14.6)	0.001^β^	11.3 (6.2–17.1)
33^rd^ and 66^th^ percentiles	8.9 and 15.8	6.4 and 12.5		7.6 and 14.6
**BMI**				
Mean (SD)	28.2 (5.4)	27.6 (5.8)	0.31^α^	28.0 (5.5)
% obese (BMI ≥30)	28.2	31.5	0.51^γ^	29.4

The multiple Cox regression showed no significant difference in time to initiation of biologic treatment for women versus men with a HR of 1.20 (CI 95% 0.94 to 1.52), table 3. Patients with high PASI were more likely to receive biologic treatment; the HR for medium PASI was 2.12 (CI 95% 1.48 to 3.05) and for high PASI 7.91 (CI % 5.65 to 11.05) when compared to the reference category of low PASI. Further, there was a significant trend that younger patients were more likely to be treated with a biologic agent. The HR for age group 10–20 years was 2.10 (CI 95% 1.12 to 3.93), age group 21–30 1.29 (CI 95% 0.83 to 2.02), age group 41–50 0.93 (CI 95% 0.67 to 1.31), age group 51–60 0.66 (CI 95% 0.46 to 0.93), age group 61–70 0.25 (CI 95% 0.14 to 0.46), age group 71+0.25 (CI 95% 0.14 to 0.46), compared to the reference age group of 31–40. BMI was not an explanatory factor regarding initiation of biologic treatment in PsoReg (table 3). The HR for psoriatic arthritis was 1.97 (CI 95% 1.57 to 2.48) which indicated that an on-going arthritis almost doubled the chances of receiving biologic treatment.

**Table 3:** The chance (hazard) to receive biologic treatment analyzed by multiple Cox regression with time to biologic initiation as outcome.^1.^

Covariate		Unadjusted HR (95% CI)	P-value	Adjusted HR (95% CI)	P-value
**Sex**	Female	0.83 (0.66 to 1.03)	0.09	1.20 (0.94 to 1.52)	0.15
**Age**	10–20	2.35 (1.31 to 4.20)	0.004	2.10 (1.12 to 3.93)	0.02
	21–30	1.13 (0.73 to 1.73)	0.59	1.29 (0.83 to 2.02)	0.26
	31–40	***Reference***			
	41–50	0.90 (0.65 to 1.24)	0.52	0.93 (0.67 to 1.31)	0.69
	51–60	0.65 (0.47 to 0.91)	0.01	0.66 (0.46 to 0.93)	0.02
	61–70	0.35 (0.25 to 0.51)	<.001	0.41 (0.27 to 0.60)	<.001
	71+	0.19 (0.11 to 0.34)	<.001	0.25 (0.14 to 0.46)	<.001
**BMI**	Underweight	1.60 (0.81 to 3.17)	0.18	1.21 (0.58 to 2.53)	0.61
	Normal	***Reference***			
	Overweight	1.14 (0.87 to 1.47)	0.34	1.31 (0.99 to 1.73)	0.06
	Obese	1.29 (0.98 to 1.70)	0.07	1.24 (0.92 to 1.67)	0.16
**Psoriatic arthritis**	Diagnosis	2.08 (1.66 to 2.59)	<.001	1.97 (1.57 to 2.48)	<.001
**PASI**	Low	***Reference***			
	Medium	2.15 (1.52 to 3.04)	<.001	2.12 (1.48 to 3.05)	<.001
	High	8.50 (6.25 to 11.57)	<.001	7.91 (5.65 to 11.05)	<.001

1 All factors in the table were included as co-variates; the model was stratified by calendar-year.

**Figure 2:**
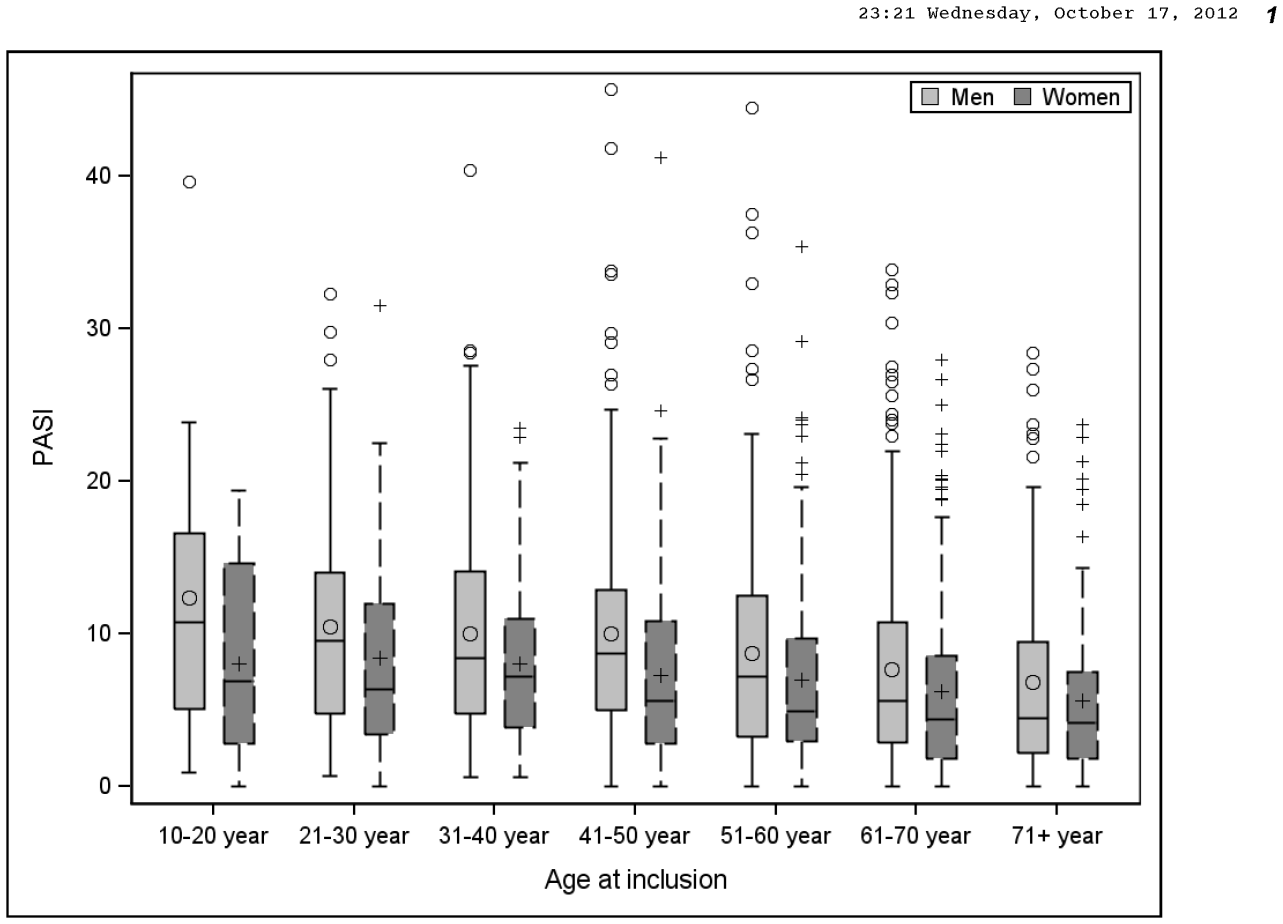
PASI at baseline for sex and different age groups.

The observed median for PASI at baseline was higher for men in all of the age groups compared with women. There were also more extreme high values among men compared to women, figure 2.

**Figure 3:**
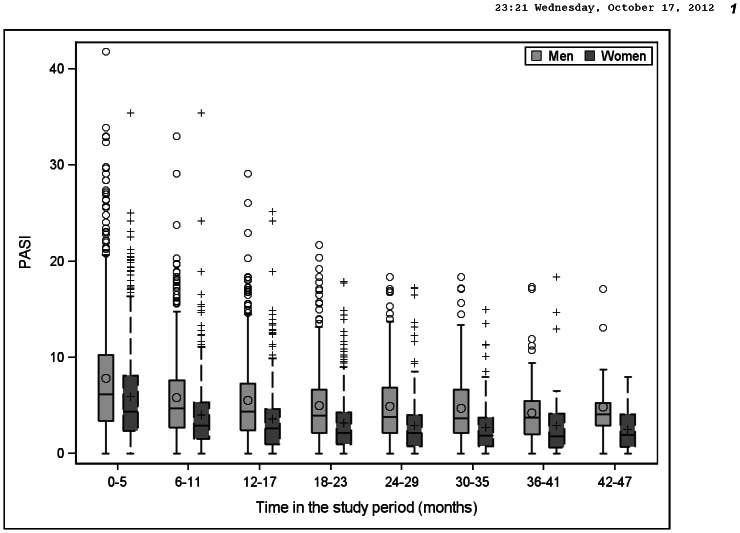
PASI at different time periods in PsoReg. Use or not of biologic treatment is not included in this graph.

The PASI-score decreased for both men and women over time after inclusion in the register, but men had a higher median PASI-score in each time period compared to women, figure 3.

## Discussion

We were able to exclude sex as an independent factor for the allocation of biologic therapy in PsoReg. Men had, at enrolment, a more severe psoriasis than women, consistent across all age groups (figure 2) and consistently at different follow-up times (figure 3). The outliers in figures 2 and 3 indicate further that men tend to have higher extreme PASI values compared to women. We observed that the degree of disease severity was the most important factor for initiation of biologic treatment. Taken together, these results indicate that the overrepresentation of men in PsoReg treated with biologics possibly mirrors their higher disease burden measured by the PASI, rather than a discrimination against women regarding access to biologic treatment.

The assessment of the severity of psoriasis (PASI) is an - ideally - objective interpretation of the disease severity made by the clinicians, but there is a risk of different interpretations depending on the physiciańs personal perception and experience, which could lead to some degree of misclassification regarding the PASI scores. There is however no obvious reason to believe that this misclassification would be systematic or differential. The selection of patients that are enrolled in PsoReg are limited to those who are suffering from moderate to severe psoriasis, and who have been referred to a specialist in dermatology. Thereby the generalizability of this study is limited to patients with moderate to severe disease treated by a specialist. However, the patients in PsoReg are probably a representative sample of patients that are most likely to receive biologic therapies. A preliminary analysis of the prescription of biologics for psoriasis in Sweden based on the national prescribed drug register and patient registry (manuscript in preparation) corroborated the overrepresentation of men receiving biologics observed in PsoReg, indicating that PsoReg is not biased in terms of sex distribution.

The sex ratio of the prevalence of a given disease is easy to analyse. On the contrary, the documentation of an unbalanced severity distribution between the sexes hidden within a balanced prevalence is difficult to verify and requires robust, highly detailed data. About 59% of patients in the Swedish registry for systemic psoriasis treatment PsoReg are men and 63% of those who started biologics were men. Registries for systemic psoriasis treatment in Europe show an even more dramatic dominance of men, ranging from 68% in the Netherlands to 60% in Germany [10]. Already in 2000 Hotard *et al* observed that only 39% of patients receiving systemic treatments were women, although more women consulted a physician for their psoriasis. Later Nyberg *et al* and White *et al* noted that men receive more UV- and systemic treatment [13], [14]. As these studies did not include data on disease severity, they were difficult to interpret. PsoReg however gave us the unique chance for a nationwide analysis not only considering disease activity at a given point of time, but longitudinally over time. Furthermore, PsoReg contains highly detailed data, which allows controlling for potential confounding. The analysis from the multiple Cox proportional regression gives consistent results with intuitively interpretable gradients in all significant variables. With the exception of sex, the HR:s did not differ substantially between the unadjusted and the adjusted Cox regression (table 3). It should be noted that the decision to start biological treatment is made by the doctor in dialogue with the patient, and this process is not captured in the registry. We can therefore not exclude a potential bias deriving from this undocumented dialogue.

It may be expected that younger patients are elected for biologic therapy, both because of concerns of possible side-effects in the elderly, but also because younger patients can be hypothesized to gain more from an effective treatment – e.g. the possibility to avoid sick-leave - than older patients would. It is also expected that psoriatic patients with arthritic involvement be selected for biologic treatment, since such therapy in patients with rheumatoid arthritis is proven very effective.

Traupe et al. have described a male dominance in psoriasis in terms of genomic Imprinting; children from fathers with psoriasis developed significantly more often psoriasis as children from mothers with psoriasis [18]. It is possible that the genes underlying this imprinting even contribute to a higher disease activity in males. In general, if we consider the known sex-based differences of both the innate and adapted immune responses [19], it is not surprising that an immunological disease such as psoriasis shows sex-dependent variations in disease severity.

In the present analysis, the factor of interest was the biologic expression of sex and psoriasis, and this is a striking simplification of a complex reality. Gender, i.e. the social aspects of being men and women in a given context, is not within the scope of this study. Likewise, the effect of moderate to severe psoriasis on the health related quality of life has not been studied here. It is possible that a focus on gender and health related quality of life may show that a discrimination against women exists.

This article shows that women *per se* are not discriminated regarding access to expensive biologic therapy, but rather suggests that the underlying disease severity differs between the sexes. Further studies of psoriasis, including Randomized Clinical Trials (RCTs) should take this difference into account.
